# Sodium-glucose cotransporter 2 inhibitors induce anti-inflammatory and anti-ferroptotic shift in epicardial adipose tissue of subjects with severe heart failure

**DOI:** 10.1186/s12933-024-02298-9

**Published:** 2024-06-28

**Authors:** Barbora Judita Kasperova, Milos Mraz, Petr Svoboda, Daniel Hlavacek, Helena Kratochvilova, Istvan Modos, Nikola Vrzackova, Peter Ivak, Petra Janovska, Tatyana Kobets, Jakub Mahrik, Martin Riecan, Lenka Steiner Mrazova, Viktor Stranecky, Ivan Netuka, Tomas Cajka, Ondrej Kuda, Vojtech Melenovsky, Sona Stemberkova Hubackova, Martin Haluzik

**Affiliations:** 1 Diabetes Centre, Institute for Clinical and Experimental Medicine, Videnska 1958/9, 140 21 Prague, Czech Republic; 2 First Faculty of Medicine, Charles University in Prague, Katerinska 1660/32, 121 08 Prague, Czech Republic; 3 Institute of Medical Biochemistry and Laboratory Diagnostics, First Faculty of Medicine, Charles University and General University Hospital, U Nemocnice 499/2, 128 08 Prague, Czech Republic; 4 Centre for Experimental Medicine, Institute for Clinical and Experimental Medicine, Videnska 1958/9, 140 21 Prague, Czech Republic; 5 Department of Biochemistry and Microbiology, University of Chemistry and Technology Prague, Technicka 5, 166 28 Prague, Czech Republic; 6 Department of Cardiac Surgery, Institute for Clinical and Experimental Medicine, Videnska 1958/9, 140 21 Prague, Czech Republic; 7 Third Faculty of Medicine, Charles University in Prague, Ruska 87, 100 00 Prague, Czech Republic; 8 Department of Informatics, Institute for Clinical and Experimental Medicine, Videnska 1958/9, 140 21 Prague, Czech Republic; 9 Department of Adipose Tissue Biology, Institute of Physiology of the Czech Academy of Sciences, Videnska 1083, 142 00 Prague, Czech Republic; 10 Department of Metabolomics, Institute of Physiology of the Czech Academy of Sciences, Videnska 1083, 142 00 Prague, Czech Republic; 11 Department of Cardiac Anesthesia, Institute for Clinical and Experimental Medicine, Videnska 1958/9, 140 21 Prague, Czech Republic; 12 Department of Metabolism of Bioactive Lipids, Institute of Physiology of the Czech Academy of Sciences, Videnska 1083, 142 00 Prague, Czech Republic; 13 Research Unit for Rare Diseases, Department of Pediatrics and Inherited Metabolic Disorders, First Faculty of Medicine, Charles University and General University Hospital, Ke Karlovu 455/2, 128 08 Prague, Czech Republic; 14 Department of Cardiology, Institute for Clinical and Experimental Medicine, Videnska 1958/9, 140 21 Prague, Czech Republic

**Keywords:** Sodium-glucose cotransporter 2 inhibitors, Heart failure, Inflammation, Adipose tissue, Ether lipids

## Abstract

**Background:**

Sodium-glucose cotransporter 2 inhibitors (SGLT-2i) are glucose-lowering agents used for the treatment of type 2 diabetes mellitus, which also improve heart failure and decrease the risk of cardiovascular complications. Epicardial adipose tissue (EAT) dysfunction was suggested to contribute to the development of heart failure. We aimed to elucidate a possible role of changes in EAT metabolic and inflammatory profile in the beneficial cardioprotective effects of SGLT-2i in subjects with severe heart failure.

**Methods:**

26 subjects with severe heart failure, with reduced ejection fraction, treated with SGLT-2i versus 26 subjects without treatment, matched for age (54.0 ± 2.1 vs. 55.3 ± 2.1 years, n.s.), body mass index (27.8 ± 0.9 vs. 28.8 ± 1.0 kg/m^2^, n.s.) and left ventricular ejection fraction (20.7 ± 0.5 vs. 23.2 ± 1.7%, n.s.), who were scheduled for heart transplantation or mechanical support implantation, were included in the study. A complex metabolomic and gene expression analysis of EAT obtained during surgery was performed.

**Results:**

SGLT-2i ameliorated inflammation, as evidenced by the improved gene expression profile of pro-inflammatory genes in adipose tissue and decreased infiltration of immune cells into EAT. Enrichment of ether lipids with oleic acid noted on metabolomic analysis suggests a reduced disposition to ferroptosis, potentially further contributing to decreased oxidative stress in EAT of SGLT-2i treated subjects.

**Conclusions:**

Our results show decreased inflammation in EAT of patients with severe heart failure treated by SGLT-2i, as compared to patients with heart failure without this therapy. Modulation of EAT inflammatory and metabolic status could represent a novel mechanism behind SGLT-2i-associated cardioprotective effects in patients with heart failure.

**Supplementary Information:**

The online version contains supplementary material available at 10.1186/s12933-024-02298-9.

## Background

Heart failure (HF), a syndrome defined by the presence of typical symptoms (e.g. dyspnoea and fatigue) and signs (e.g. peripheral oedema or pulmonary crackles), caused by structural or functional cardiac abnormalities and accompanied by pulmonary or systemic congestion, represents one of the leading worldwide pandemics with high rates of morbidity and mortality [1]. Its prevalence is rising with the aging population and, in 2020, HF was estimated to affect 64.3 million people globally, accounting for 9.9 million years lost due to disability [2]. In developed countries, an estimated 1–2% of the adult population suffer from HF, with another 2% going undetected [3]. Observational data show a 5-year mortality of more than 60% after diagnosis of heart failure with reduced ejection fraction (HFrEF—left ventricular ejection fraction < 40%) [4]. Thus, HF has become an immense burden on both individual patients and the healthcare and social systems.

The mainstay of HF treatment has for long been the combination of angiotensin-converting enzyme inhibitors or angiotensin II receptor blockers, beta-blockers, mineralocorticoid receptor antagonists and loop diuretics, with a later addition of angiotensin receptor/neprilysin inhibitors [5]. Recently, a new class of medicaments, originally designed as oral glucose-lowering agents, the sodium-glucose cotransporter 2 inhibitors (SGLT-2i), showed surprising cardiovascular benefits in general cardiovascular outcome trials, driven in large part by the reduction of hospitalisations for HF [6–8]. These beneficial effects were further confirmed in dedicated HF trials, regardless of the presence of diabetes mellitus and also in subjects with heart failure with preserved ejection fraction (HFpEF—left ventricular ejection fraction > 50%) [9–12]. SGLT-2i work primarily by inhibiting the sodium-glucose cotransporter 2 in the renal proximal convolute tubule, leading to significantly increased excretion of glucose into urine. However, as the effect of SGLT-2i on HF improvement was present across a wide range of glomerular filtration rates and also in subjects with substantial renal impairment, glycosuric action does not seem to be the only responsible factor [13]. In fact, a number of other contributing mechanisms were proposed to explain the beneficial effects of SGLT-2i on HF, including improved haemodynamics and reduced cardiac filling pressure due to their diuretic effect, enhanced myocardial energetic efficiency due to increased supply with ketone bodies, reduction of oxidative stress and pro-inflammatory factors, changes in myocardial intracellular ion handling (especially sodium and calcium), reduction of extracellular matrix remodelling and myocardial fibrosis and, most recently, the effects on epicardial adipose tissue [14–17].

Epicardial adipose tissue (EAT) is a fat depot located between the myocardium and visceral pericardium, concentrated especially in the atrioventricular and interventricular grooves, as well as above the ventricular and atrial walls [18]. Due to the absence of a clear biological barrier (i.e. fascia) and a shared microcirculation (supplied by the coronary arteries), EAT and its products can directly affect the adjacent myocardium via paracrine and vasocrine mechanisms [19, 20]. This anatomical and functional integrity (unique to EAT among other visceral adipose tissue depots) helps to protect the myocardium under physiological conditions (e.g. protecting against hypothermia or regulating free fatty acid supply), while, on the other hand, directly deranging myocardial structure and function in the case of EAT dysfunction, usually associated with obesity and type 2 diabetes mellitus (T2DM), thus contributing to the development of cardiac diseases including HF, coronary artery disease (CAD) and atrial fibrillation [21, 22]. In HFpEF, a growing body of evidence supports its association with the expansion of EAT (typically seen in obesity), which not only mechanically impairs left ventricular (LV) distensibility and increases its filling pressure, but, via the secretion of pro-inflammatory cyto- and adipokines, contributes to myocardial fibrosis and increased intramyocellular lipid content [23–27]. Far less data are available on the relationship between EAT and HFrEF, with some studies showing reduced EAT mass and thickness in subjects with HFrEF, as compared with healthy controls or HFpEF subjects, possibly representing an early feature of cardiac cachexia [28, 29]. Interestingly, treatment with SGLT-2i was associated with reduced EAT amount in subjects with HFrEF, CAD and T2DM and obesity, although the results were not unequivocal [30–33]. However, thus far only a few human studies have tried to analyse direct metabolic, inflammatory and other molecular effects of EAT on the myocardium in HFrEF with, to our knowledge, none of them specifically covering the impact of SGLT-2i treatment.

To this end, we performed a comprehensive analysis of EAT of subjects with severe HFrEF undergoing elective heart transplantation or mechanical assist device implantation and compared it with subcutaneous adipose tissue (SAT) representing an adipose tissue depot with far anatomical and functional proximity to myocardium. The analysis combined complex metabolomics and gene expression analysis, along with immunohistochemistry and flow cytometry, to identify possible cardioprotective factors and pathways associated with SGLT-2i treatment.

## Methods

### Study subjects

This was a cross-sectional clinical trial, performed in subjects undergoing heart transplantation or mechanical circulatory support implantation for end-stage heart failure (left ventricular ejection fraction (LV EF) ≤ 30%, New York Heart Association (NYHA) III-IV, brain natriuretic peptide—BNP ≥ 200 pmol/L). Inclusion criteria were patients with severe heart failure (HF) (HF history ≥ 6 months, NYHA III–IV, LV EF < 30%, BNP ≥ 200 pmol/L, undergoing implantation of mechanical circulatory support (LVAD) or heart transplantation, minimum of 14 days of SGLT-2i treatment (only for SGLT-2i+ group). Exclusion criteria included acute coronary syndrome, acute inflammatory state, active malignancy.

Participants were divided into two groups according to the presence of SGLT-2i treatment and both groups were matched according to age, body mass index (BMI) and LV EF. To ensure the closest possible matching between our two cohorts, subjects were paired with each other, using the nearest neighbour method on propensity scores. The propensity scores were computed using logistic regression, where the outcome variable was the treatment indicator and age, BMI, BNP, LV EF were used as independent variables.

All participants signed written informed consent prior to enrolment in the study. Since the samples were taken as part of a planned surgery, the patients were not compensated. The study was approved by the Human Ethics Review Board, Institute for Clinical and Experimental Medicine (IKEM), Prague, Czech Republic (ethical approval code G-18-36) and was performed in agreement with the principles of WMA Declaration of Helsinki—Ethical Principles for Medical Research Involving Human Subjects [34].

### Surgical methods

Samples of EAT and subcutaneous adipose tissue (SAT) were taken perioperatively after 6–12 h of fasting for further analyses. All procedures were performed from median sternotomy, providing optimal access to the heart. EAT is mostly distributed as localised depots in atrioventricular and inter-ventricular grooves and atrial appendages. The EAT samples were retrieved mostly from the free wall of the right ventricle, considering the potential risk of damage to underlying structures (especially the right coronary artery). SAT samples were harvested from the lower pole of the sternotomy site, from approximately the same location in all patients. Freshly collected specimens in phosphate buffered saline (0.01 mol/L, pH 7.4; PBS; Sigma, St. Louis, MO, USA) were used for flow cytometry, and aliquots were stored at − 80 °C.

### Biochemical analysis

Blood samples were taken before the beginning of surgery after 6–12 h’ fasting. Samples were centrifuged for 10 min at 1000×*g* within 30 min after withdrawal at room temperature. Serum or plasma aliquots were subsequently stored at − 80 °C. Biochemical parameters were measured, and low-density lipoprotein cholesterol was calculated at the in-house Laboratory Methods Division by standard laboratory methods.

### Luminex and ELISA

Serum levels of cytokines like Interferon gamma (IFNγ), Interleukin (IL) 6, IL8, IL10 and Tumour Necrosis Factor α (TNFα) were measured by the multiplex assay MILLIPLEX® Human Cytokine/Chemokine Magnetic Bead Panel (HCYTOMAG-60K; Merck KGaA, Darmstadt, Germany—only 5 cytokines were selected and measured form the whole panel), using the MAGPIX system (Luminex corporate, Austin, TX, USA). Serum levels of BNP were measured by the ARCHITECT BNP (8K28 ARCHITECT BNP Reagent Kit, Abbott, Plymouth, MN, USA), using Abbott ARCHITECT c16000 (Abbott, Plymouth, MN, USA). Serum C-reactive protein (CRP) levels were measured by the Invitrogen Human CRP ELISA kit (BMS288INST; Thermo Fisher Scientific, Waltham, Massachusetts, USA).

### Flow cytometry analysis

Flow cytometry (FACS) was performed, using freshly isolated and filtered EDTA whole blood. A total amount of 100 µL of cell suspension with average ~ 1 × 10^6^ cell content was labelled by monoclonal antibodies. The samples were labelled in the dark for 30 min at 2–8 °C, and then red blood cells were lysed, using Excellyse I (Exbio Prague, a.s., Vestec, Czech Republic) according to the manufacturer’s instructions. Samples were analysed on Navios Flow Cytometer (Beckman Coulter, Brea, CA, USA). Data analysis was performed using FlowJo X 10.0.7r2 software (FlowJo, LCC, Ashland, USA). The gating strategy was as follows: doublets were excluded and T helper lymphocytes were gated according to SSC properties and CD45 positivity, and then CD4 positivity. From CD4+ gate Th2 lymphocytes were assessed as CD294+CD183−CD4+CD3+CD45+ (CRTH2 positive) cells and Th1 lymphocytes as CD294−CD183+CD4+CD3+CD45+ (CXCR3 positive). Minimal count of acquired was 100,000.

### RNA library preparation, sequencing and data analysis

#### Sample preparation and sequencing

Total RNA was isolated from tissue samples (80–100 mg) of EAT and SAT, using the RNeasy Lipid Tissue Mini Kit (Qiagen, Hilden, Germany). Its quality was checked using HS RNA Kit and measured on a 5200 Fragment Analyzer System (Agilent Technologies, Santa Clara, CA, USA). Regarding the variability of RIN (2.5–7.8), the RNA-seq library was prepared by RNA-depletion, using the KAPA RNA HyperPrep Kit with RiboErase (HMR) (Roche, Rotkreuz, Switzerland), according to the manufacturer’s instructions. Libraries were pooled and sequenced on NovaSeq 6000 system (Illumina, San Diego, CA, USA) to produce 100-basepair paired-end reads at the National Centre for Medical Genomics in Prague.

The resulting FASTQ files were subjected to QC control and trimmed, using Atropos (version1.128). Gene-level abundances were estimated, using Salmon (version 1.3) with the Ensembl gene definition version 94 [35].

#### Detection of differentially expressed genes

Differential expression (DE) analysis was performed in R environment (version 4.3.0). The analysis was performed in SGLT-2i− versus SGLT-2i+ pairs separately for both EAT and SAT. For EAT, samples from 6 subjects without SGLT-2i treatment and 6 subjects with SGLT-2i treatment were analysed. Similarly for SAT, samples from 6 subjects without SGLT-2i treatment and 7 subjects with SGLT-2i treatment were examined. In total, 12,590 expressed genes were analysed in both tissues. Identification of DE genes and calculation of log2 fold changes were performed, using the R package DESeq2 (version 1.42.0 [36]), with a standard DESeq2 pipeline using default parameters. Briefly, the read count data, pre-filtered for low-expressed genes, were normalised with the median of ratios method. Groups were compared with the Wald test, and *p*-values were adjusted with the Benjamini–Hochberg method. Only protein-coding genes were considered for further steps. Resulting DE genes were filtered for significance with soft cut-off criteria, including non-adjusted *p*-value of 0.05, the absolute rounded log_2_ fold change (log2FC) of 0.5, and the minimal baseMean of 50. The soft cut-offs corresponded to 40% of difference between compared groups. No genes met the FDR significance rate of 0.1, due to the high individual variability in compared datasets from human patients. For plotting of the input data and results, regularised logarithmic transformation (rlog) was applied.

#### Functional annotation of results using over-representation analysis

Lists of DE genes fitting the soft cut-offs were processed in the clusterProfiler R package (version 4.10.0 [37]), using over-representation analysis (ORA) and data from Gene Ontology [38, 39], KEGG [40], Reactome [40], WikiPathways [41, 42] and Disease Ontology [43] databases. Resulting ORA *p*-values were corrected with the Benjamini–Hochberg method. Terms and pathways with the adjusted *p*-value less than 0.05 were selected.

### Metabolomics and lipidomics

Metabolites were extracted using a biphasic solvent system of cold methanol, methyl *tert*-butyl ether, and water. In more detail, 10–25 mg of adipose tissue samples were homogenised (1.5 min) with 275 µL methanol and 275 µL 10% methanol, both containing internal standards using a grinder. Then, 1 mL of methyl *tert*-butyl ether with internal standard was added, and the tubes were shaken (1 min) and centrifuged (16,000 rpm, 5 min, 4 °C) [44, 45].

In total, 7 different Liquid Chromatography–Mass Spectrometry (LC–MS) platforms were used for metabolomic and lipidomic profiling: (i) lipidomics of high-abundant triacylglycerols in positive ion mode; (ii) lipidomics of low-abundant triacylglycerol estolides in positive ion mode; (iii) lipidomics of minor polar lipids in positive ion mode; (iv) lipidomics of minor polar lipids in negative ion mode; (v) metabolomics of polar metabolites in positive ion mode (BEH Amide platform); (vi) metabolomics of polar metabolites in negative ion mode (HSS T3 platform); and (vii) targeted analysis of fatty acid esters of hydroxy fatty acid species in negative ion mode.

#### Untargeted lipidomics

For profiling of high-abundant triacylglycerol and low-abundant triacylglycerol estolides, 10 µL of the upper organic phase was collected and evaporated. Then, the dry extracts were resuspended, using 1 mL methanol containing 12-[(cyclohexylamino)carbonyl]amino]-dodecanoic acid (CUDA) internal standard, shaken (30 s), centrifuged (16,000 rpm, 2 min, 4 °C), and used for lipidomic Electrospray ionisation in the positive mode platforms with different injection volumes and mass range collected. For profiling of minor polar lipid species in positive and negative ion modes, 100 µL of the upper organic phase was collected and evaporated. Then, the dry extracts were resuspended using 80% methanol with CUDA internal standard, shaken (30 s), centrifuged (16,000 rpm, 5 min, 4 °C), and used for LC–MS analysis.

#### Untargeted metabolomics

For profiling of polar metabolites, an aliquot of 70 µL of the bottom aqueous phase was collected and evaporated. Then, the dry extracts were resuspended in 70 µL of an acetonitrile/water (4:1) mixture with CUDA and Val–Tyr–Val internal standards, shaken (30 s), centrifuged (16,000 rpm, 5 min, 4 °C), and analysed using the HILIC metabolomics platform. In addition, another 70 µL of the bottom aqueous phase was mixed with 210 µL of an isopropanol/acetonitrile (1:1) mixture, shaken (30 s), centrifuged (16,000 rpm, 5 min, 4 °C), and the supernatant was evaporated. Then, the dry extracts were resuspended in 5% methanol/0.2% formic acid with CUDA and Val–Tyr–Val internal standards, shaken (30 s), centrifuged (16,000 rpm, 5 min, 4 °C), and analysed using the HSS T3 metabolomics platform.

The LC–MS systems consisted of a Vanquish UHPLC System (Thermo Fisher Scientific, Waltham, Massachusetts, USA) coupled to a Q Exactive Plus mass spectrometer (Thermo Fisher Scientific, Waltham, Massachusetts, USA).

LC–MS data from metabolomic and lipidomic profiling were processed through MS-DIAL v. 4.80 software [44]. Metabolites were annotated, using in-house retention time–*m*/*z* library and MS/MS libraries available from various sources (NIST20, MoNA, LipidBlast). Raw data were filtered using blank samples, serial dilution samples, and quality control pool samples with relative standard deviation < 30%, normalised using the LOESS approach, utilising quality control pool samples injected regularly between 10 actual samples, and further normalised using the amount of adipose tissue samples taken for analysis. Samples were randomised across the platform run.

### Immunohistochemistry

Adipose tissue, EAT and SAT, was collected during surgery and was frozen immediately. Tissue was fixed overnight with 2% formaldehyde, washed extensively with PBS, incubated in dimethyl sulfoxide for 1 h at room temperature and subsequently in 30% sucrose overnight at 4 °C. After this step, tissue was incubated in an increasing concentration of OCT mounting medium (20%, 50%, 70%) and frozen in 100% OCT (Sigma, St. Louis, MO, USA). Cryo-sections (4 µm) were permeabilised by a combination of dimethyl sulfoxide (15 min at room temperature; Sigma, St. Louis, MO, USA) and 0.5% saponin (Thermo Fisher, Waltham, Massachusetts, USA) for 15 min at room temperature. After washing with PBS, sections were incubated in 10% foetal bovine serum (Sigma, St. Louis, MO, USA) diluted in PBS for 30 min to block unspecific signals. After this step, sections were stained with diluted primary antibody anti-F4/80 (70076S, Cell Signaling Technology, Danvers, Massachusetts, USA) or Tomm20 (EPR15581-54, Abcam, Cambridge, Great Britain) diluted 1:100 in PBS (overnight incubation at 4 °C). Samples were washed with PBS and incubated with secondary antibody Alexa 488 goat anti-rabbit (A11034, Thermo Fisher, Waltham, Massachusetts, USA), diluted 1:1000 in PBS. To counterstain nuclei, coverslips were mounted in Mowiol containing 4’,6-diamidino-2-phenylindole (DAPI; Sigma St. Louis, MO, USA), and the signal was detected, using the Leica SP8 FLIM confocal microscope (Leica Microsystems, Mannheim, Germany).

### Quantitative reverse transcription polymerase chain reaction (RT-qPCR)

Tissue (1–2 mm^3^) was homogenised on the MagNA Lyser Instrument (1 × 30 s at 5 600 rpm) (Roche Diagnostics GmbH, Mannheim, Germany). RNA was immediately extracted from homogenised tissue, using the Qiagen RNeasy Lipid Tissue Mini Kit (Qiagen, Hilden, Germany). The RNA concentration was determined from absorbance at 260 nm on the NanoDrop™ 2000/2000c spectrophotometer (Thermo Scientific, Waltham, Massachusetts, USA). Reverse transcription was performed, using random hexamer primers according to the manufacturer’s protocol of the High-Capacity cDNA Reverse Transcription Kits (Thermo Fisher Scientific, Waltham, Massachusetts, USA). Gene expression was performed on a ViiA 7 Real-Time PCR System (Thermo Fisher Scientific, Waltham, Massachusetts, USA), with specific primers (Table 1) purchased from Sigma, St. Louis, MO, USA. Relative quantity of cDNA was estimated by the 2^−ΔΔCT^ method; data were normalised to the housekeeping *β-actin* gene.

**Table 1:** List of primers for RT-qPCR

Gene	Forward	Reverse
*ACTB*	CCAACCGCGAGAAGATGA	CCAGAGGCGTACAGGGATAG
*ADGRE1*	TGTGACGTTGGACTTGGTAGCC	GGAGACAAAAGCCACACCAGTG
*TF*	TCAGCAGAGACCACCGAAGACT	GACCACACTTGCCCGCTATGTA

ACTB, β-actin; ADGRE1, adhesion G protein-coupled receptor E1; TF, transferrin

### Statistical analysis

Statistical analysis was performed, and graphs were drawn using SigmaPlot 13.0 software (SPSS Inc., Chicago, IL, USA). The results are expressed as mean ± standard error of the mean (SEM). Normality of all data was assessed by the Shapiro–Wilk test. One-way ANOVA followed by the Holm–Sidak test and an unpaired t-test or Wilcoxon Signed-Rank test were used for the assessment of intra- and intergroup differences, as appropriate. MetaboAnalyst 5.0 [46] was used to explore the metabolomics and lipidomics data (log10 transformation and data scaling) and Plotly 5.15 [47] was used to create the plots. Achieving *p*-value < 0.05 was deemed statistically significant.

## Results

### Baseline characteristics of study subjects

Baseline characteristics of study patients are presented in Table 2. Patients (*n* = 52; males = 47, females = 5) undergoing heart transplantation or mechanical circulatory support implantation for end-stage heart failure (EF ≤ 30%, New York Heart Association (NYHA) III-IV, brain natriuretic peptide—BNP ≥ 200 pmol/L) were divided into two groups (*n* = 26), according to the presence of SGLT-2i treatment (empagliflozin or dapagliflozin). No differences between the untreated (SGLT-2i−) and SGLT-2i treated (SGLT-2i+) patients in any of anthropometric and clinical characteristics were present, including age, body mass index (BMI), LV EF left ventricular end-diastolic diameter (LVEDd) and serum BNP. Also, no differences were seen in CRP, lipid parameters, and fasting glucose, while HbA_1c_ and non-esterified fatty acids were increased in the SGLT-2i+ group, reflecting the higher number of patients with T2DM in this group (Table 2).

**Table 2:** Anthropometric, biochemical and hormonal parameters of study subjects

Parameter		SGLT-2i− group	SGLT-2i+ group	*p*-value
Number of subjects (*n*) (m/f)		26 (23/3)	26 (24/2)	> 0.999
SGLT-2i therapy (days)		–	258 ± 83	–
Age (years)		55.3 ± 2.1	54.0 ± 2.1	0.663
T2DM (*n*, %)		9 (34.6)	19 (73.1)*	0.012
BMI (kg/m^2^)		28.8 ± 1.0	27.8 ± 0.9	0.461
LV EF (%)		23.2 ± 1.7	20.7 ± 0.5	0.169
LVEDd (mm)		66.8 ± 2.7	70.2 ± 2.2	0.334
BNP (ng/L)		1247 ± 201	1123 ± 206	0.668
CRP (mg/L)		5.05 ± 3.65	4.95 ± 7.56	0.991
AST (µkat/L)		0.77 ± 0.13	0.65 ± 0.12	0.501
ALT (µkat/L)		0.77 ± 0.09	1.23 ± 0.47	0.345
Total cholesterol (mmol/L)		3.26 ± 0.23	3.30 ± 0.23	0.903
HDL (mmol/L)		0.81 ± 0.06	0.77 ± 0.04	0.582
LDL (mmol/L)		1.90 ± 0.18	1.80 ± 0.17	0.688
Triacylglycerols (mmol/L)		1.22 ± 0.09	1.55 ± 0.20	0.141
Fasting blood glucose (mmol/L)		6.60 ± 0.51	7.29 ± 0.54	0.357
HbA_1c_ (mmol/mol)		43.39 ± 1.23	52.31 ± 2.74*	0.005
Non-esterified fatty acids (mmol/L)		0.8 ± 0.1	1.3 ± 0.1*	0.001
Arterial hypertension (*n*, %)		12 (46.2)	9 (34.6)	0.572
Coronary artery disease (*n*, %)		13 (50.0)	12 (46.2)	> 0.999
Dyslipidaemia (*n*, %)		15 (57.7)	16 (61.5)	> 0.999
Medication:				
ACEi/ARB		6 (23.1)	6 (23.1)	> 0.999
ARNI		12 (46.5)	15 (57.7)	0.579
β-blockers		19 (73.1)	21 (80.8)	0.743
MRA		21 (80.8)	24 (92.3)	0.419
Digoxin		6 (23.1)	8 (30.8)	0.755
Statins		14 (53.8)	18 (69.2)	0.393

Data are expressed as mean ± SEM

ACEi, angiotensin-converting-enzyme inhibitor; ALT, alanine Aminotransferase; ARB, angiotensin II receptor blocker; ARNI, angiotensin receptor neprilysin inhibitor; AST, aspartate aminotransferase; BNP, brain natriuretic peptide; BMI, body mass index; CRP, C-reactive protein; HbA_1c_, haemoglobin A_1c_; HDL, high-density lipoprotein; LDL, low-density lipoprotein; LVEDd, left ventricular end-diastolic diameter; LV EF, left ventricular ejection fraction; MRA, mineralocorticoid receptor antagonist; T2DM, type 2 diabetes mellitus

*p < 0.05 SGLT-2i+ versus SGLT-2i− group

### SGLT-2i therapy improves the metabolism and cellular processes of adipose tissue

**Fig. 1:**
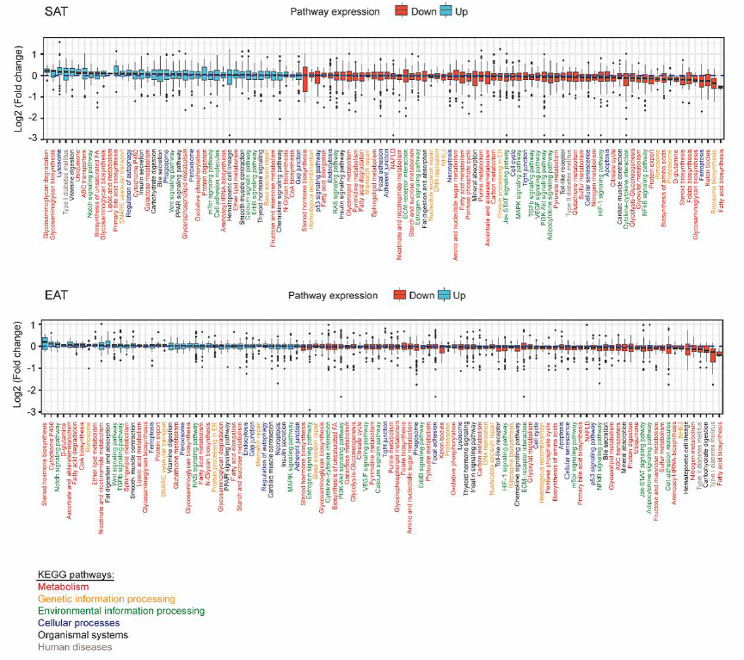
SGLT-2i treatment improves the metabolism and cellular processes of adipose tissue. EAT and SAT from SGLT-2i− and SGLT-2i+ patients were subjected to gene expression analysis, using RNAseq method. Changes of the 102 KEGG pathways in SGLT-2i+ to SGLT-2i− patients were detected. Data were utilised by the DESeq2 package in R language (version 4.2) to analyse the log fold changes of genes. The analysis was performed separately for EAT, where 6 SGLT-2i− and 6 SGLT-2i+ subjects were analysed, and for SAT, where 6 SGLT-2i− and 7 SGLT-2i+ subjects were analysed. In both tissues, a total of 2 723 genes were analysed. The log fold changes were calculated by using the DESeq2 package with the default settings and without effect size shrinkage. The resulting boxplots depict all the log fold changes within pathways, with no filtering for statistical significance. The pathways within the boxplots were sorted, based on the median of the log fold change

**Fig. 2:**
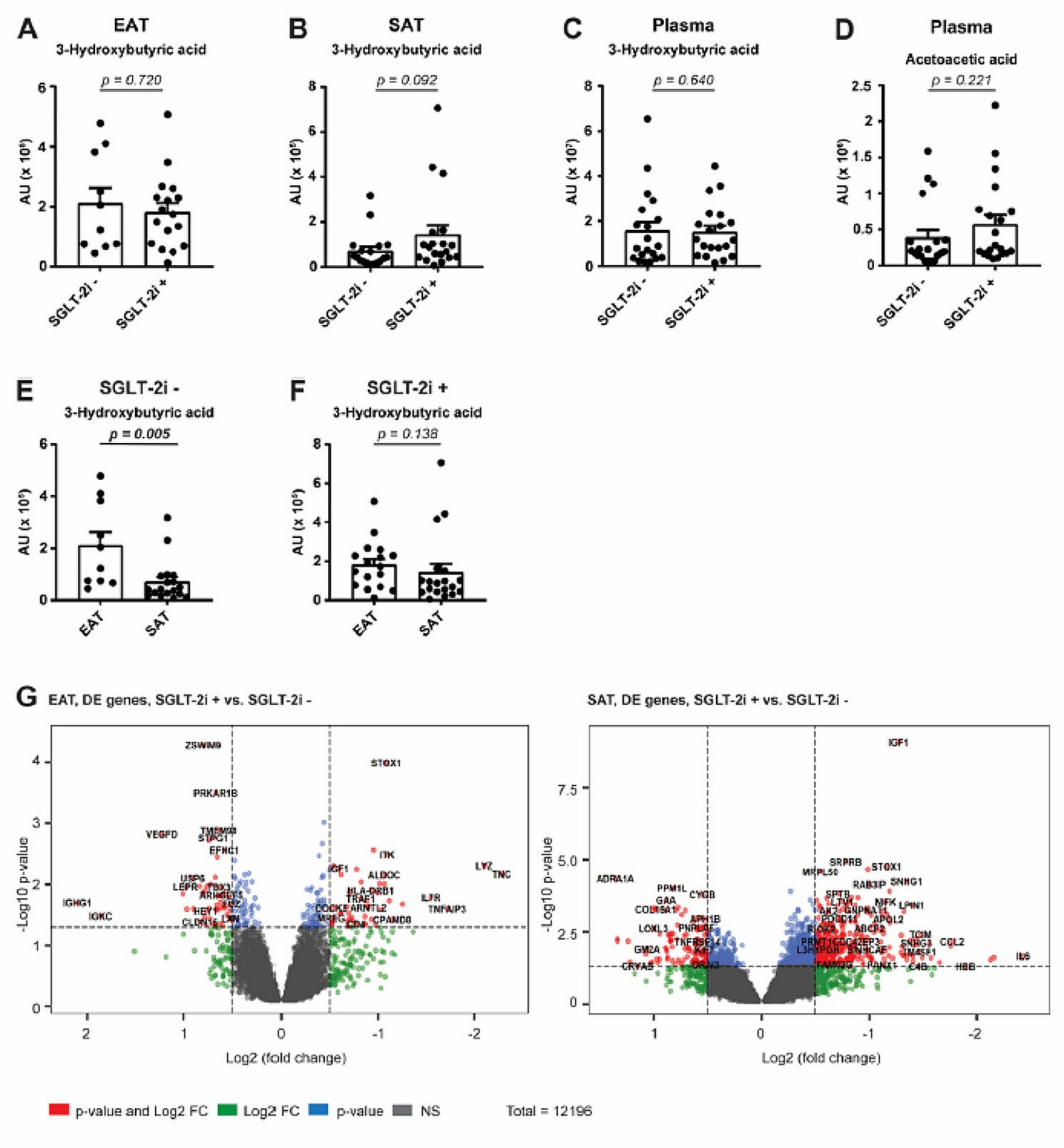
**S**GLT-2i affects ketone bodies in SAT. Level of 3-hydroxybutyric acid in **A** EAT, **B** SAT and **C** plasma and **D** level of acetoacetic acid in plasma of SGLT-2i− or SGLT-2+ patients was determined by metabolomic analysis (EAT: SGLT-2i− *n* = 10; SGLT-2i+ *n* = 17. SAT: SGLT-2i− *n* = 18; SGLT-2i+ *n* = 19. Plasma: SGLT-2i− *n* = 20; SGLT-2i+ *n* = 20.) Changes of 3-hydroxybutyric acid in EAT and SAT of **E** SGLT-2i− and **F** SGLT-2i+ patients were determined by metabolomic analysis. Data are expressed as mean ± SEM; * *p* < 0.05 with FDR correction. **G** Volcano plot represents the changes in gene expression in EAT or SAT of SGLT-2i+ to SGLT-2i− patients. Dashed lines mark the area within *p*-value < 0.1 and fold change > 1.2. Red dots represent statistically and biologically significant changes; blue dots represent statistically significant changes without biological relevance; green dots represent biologically relevant changes without statistical significance; grey dots are without statistical and biological relevance

To analyse the effects of SGLT-2i, we performed differential expression analysis in EAT and SAT from SGLT-2i+ compared to SGLT-2i− patients (Fig. 1). In both tissues, SGLT-2i reduced fatty acid biosynthesis, which in EAT was accompanied by increased fatty acid degradation. Surprisingly, in both tissues we observed decreased transcription of genes associated with biosynthesis of ketone bodies. However, metabolomic analysis revealed no changes in levels of 3-hydroxybutyric acid, a component of ketone bodies, in EAT or SAT between the SGLT-2i+ and SGLT-2i− group (Fig. 2A, B). Also, comparable levels of 3-hydroxybutyric acid and acetoacetic acid, another component of ketone bodies, were detected in plasma of SGLT-2i+ as well as SGLT-2i− patients (Fig. 2C, D). Strikingly, a direct comparison between EAT and SAT showed markedly increased 3-hydroxybutyric acid in EAT of SGLT-2i− subjects, compared with SAT, while this difference was largely nullified in the SGLT-2i+ group, mainly due to higher concentrations of 3-hydroxybutyric acid in SAT, whereas in EAT the levels were comparable with SGLT-2i− subjects (Fig. 2E, F). These findings suggest a different regulation of ketogenesis in EAT and SAT of subjects with severe heart failure and, even more interestingly, a different effect of SGLT-2i treatment on ketogenesis in these two adipose tissue depots.

Detailed analysis of gene expression showed that SGLT-2i treatment has different effects on the two adipose tissue depots. Greater changes were observed in SAT compared to EAT (Figs. 1 and 2G). In SAT, SGLT-2i treatment primarily resulted in notable changes in gene expression related to lipid and carbohydrate metabolism. Up-regulation of genes involved in metabolic pathways associated with lipid and fatty acid degradation was observed, whereas pathways involved in lipid synthesis showed down-regulation. In addition, a reduction in the expression of genes associated with pro-inflammatory pathways and, to some extent, signalling pathways linked to senescence and cell cycle arrest were evident in SAT. Similarly to SAT, in EAT SGLT-2i treatment resulted in down-regulation of gene expression associated with cell cycle inhibition, senescence, and programmed cell death (such as apoptosis, ferroptosis, or necroptosis). This phenomenon was accompanied by a decrease in inflammatory pathways, including the Janus kinase/signal transduction and transcription activation (JAK/STAT) pathway or the Nuclear Factor kappa B (NF-κB) pathway. In addition, there was an up-regulation in signalling pathways linked with tissue repair and healing, such as the Transforming Growth Factor β (TGFβ) pathway or Wingless-related integration site (WNT) pathway, and in the synthesis of ether lipids, which might contribute to the alleviation of symptoms of cardiovascular damage.

### SGLT-2i reduce inflammation in adipose tissue

Pro-inflammatory cytokines play a crucial role in adipose tissue inflammation and the development of metabolic diseases. Differential expression analysis revealed significant down-regulation and showed a significantly decreased amount of transcripts for *IL6*, *IL1R1*, *IL1RAP*, *CCL2*, *CXCL2* and *TNFAIP3* in SAT in SGLT-2i+ patients (Table 3). A similar trend of reduced gene expression was observed in EAT, but only *TNFAIP3* reached statistical significance (Table 3).

**Table 3:** Changes of selected pro-inflammatory cytokines in EAT and SAT of study subjects

Symbol	Gene name	EAT		SAT	
		Log2 FC	*p*-value	Log2 FC	*p*-value
*IL6*	Interleukin 6 (interferon, beta 2)	− 0.69	0.45	− 3.42	1.4 × 10^−3^
*CCL2*	Chemokine (C–C motif) ligand 2	− 0.21	0.50	− 2.65	0.5 × 10^−3^
*CSF2RA*	Colony stimulating factor 2 receptor, alpha, low-affinity (granulocyte-macrophage)	− 0.72	0.07	ND	
*CXCL2*	Chemokine (C–X–C motif) ligand 2	− 0.70	0.17	− 1.71	3.5 × 10^−2^
*CXCR4*	Chemokine (C–X–C motif) receptor 4	− 0.52	0.29	ND	
*IGF1*	Insulin like growth factor 1	− 0.35	0.21	− 1.67	4.5 × 10^−17^
*IL1R1*	Interleukin 1 receptor type 1	ND		− 1.42	4.5 × 10^−3^
*IL1RAP*	Interleukin 1 receptor accessory protein	ND		− 1.33	1.7 × 10^−2^
*TNFAIP3*	TNFα induced protein 3	− 1.27	0.02	− 1.73	2.5 × 10^−2^
*TRAF1*	TNF receptor associated factor 1	− 0.40	0.29	ND	

RNA-seq analysis was performed on samples of adipose tissue from subjects treated with SGLT-2i− or SGLT-2i+. Data are presented as the log fold changes of genes. EAT: SGLT-2i− *n* = 6; SGLT-2i+ *n* = 6. SAT: SGLT-2i− *n* = 6; SGLT-2i *n* = 7

SGLT-2i+ versus SGLT-2i− group; ND, not detected; FC, fold change

To complement the results of gene expression analysis, serum levels of pro-inflammatory factors including TNFα, IL6 and INFγ were measured as well, showing an all-over trend to reduced values in the SGLT-2i+ group, although the difference did not reach statistical significance (Table 4).

**Table 4:** Plasma levels of selected cytokines in study subjects

Parameter	SGLT-2i− group	SGLT2-i+ group	*p*-value
TNFα (pg/mL)	10.41 ± 1.50	8.34 ± 1.60	0.352
IL6 (pg/mL)	4.44 ± 2.48	2.34 ± 0.61	0.427
INFγ (pg/mL)	25.97 ± 5.04	20.44 ± 5.46	0.338
IL8 (pg/mL)	7.88 ± 2.19	5.65 ± 1.77	0.438
IL10 (pg/mL)	16.30 ± 2.07	14.75 ± 1.72	0.570

Data are expressed as mean ± SEM. SGLT-2i− *n* = 20; SGLT-2i+ *n* = 20

IL6, interleukin 6; IL8, interleukin 8; IL10, interleukin 10; INFγ, interferon γ; TNFα, tumour necrosis factor α

### SGLT-2i decrease accumulation of immune cells in EAT

**Fig. 3:**
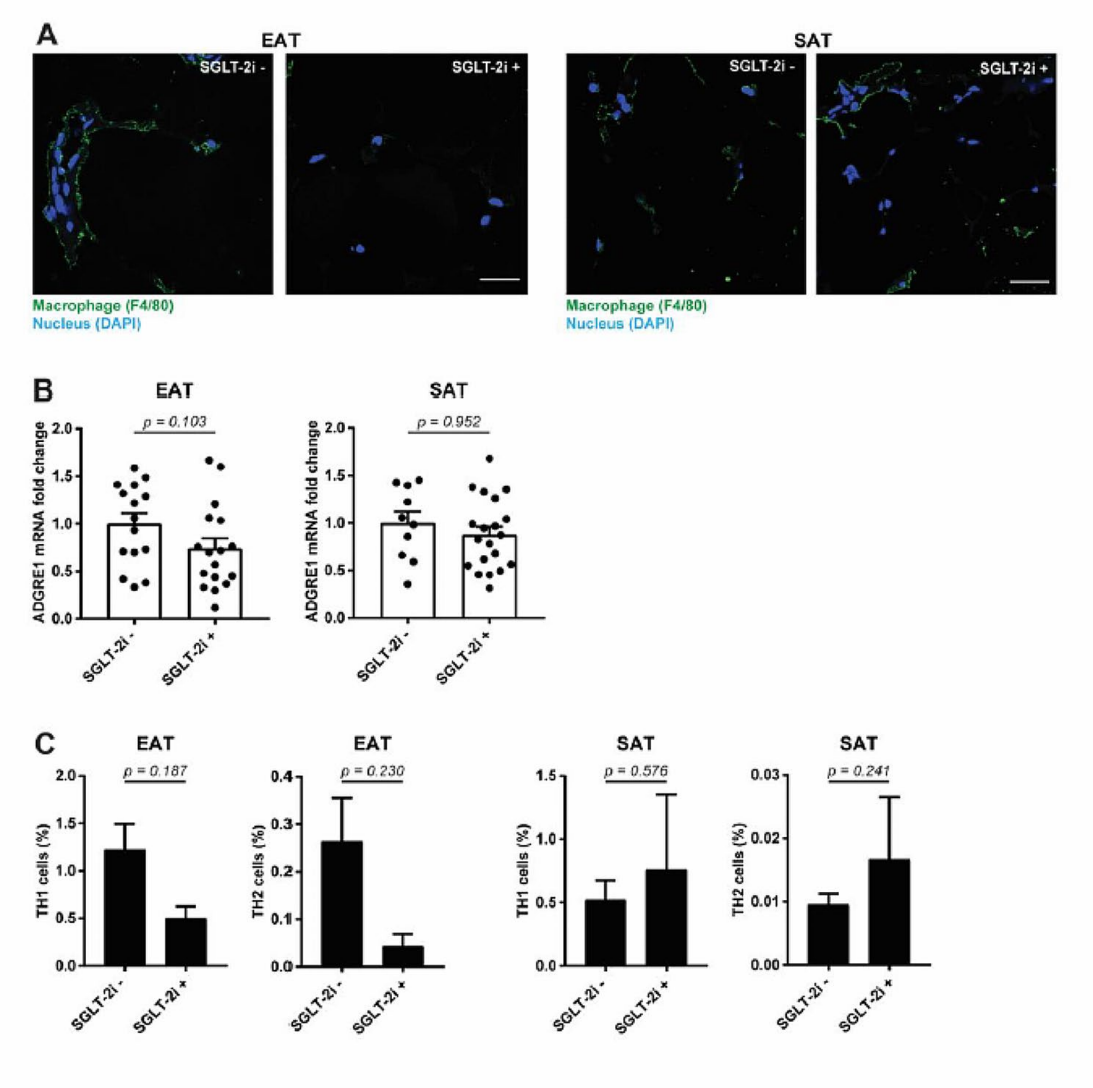
SGLT-2i decrease accumulation of immune cells in EAT. Presence of immune cells in EAT and SAT of SGLT-2i− and SGLT-2i+ patients was evaluated. **A** Macrophages were detected by F4/80 immunofluorescent staining with DAPI denoting cell nuclei (the bar indicates 50 μm), or **B** by expression of *ADGRE1* gene using RT-qPCR (EAT: SGLT-2i− *n* = 15; SGLT-2i+ *n* = 17. SAT: SGLT-2i− *n* = 17; SGLT-2i+ *n* = 25). **C** Levels of TH1 and TH2 lymphocytes were determined in EAT and SAT by FACS (SGLT-2i− *n* = 10; SGLT-2i+ *n* = 3). Data are expressed as mean ± SEM

Increased immune cell infiltration into adipose tissue contributes to the development of local/systemic inflammation and increases the risk of cardiovascular damage. Macrophages in particular represent one of the biggest producers of pro-inflammatory molecules. Immunohistochemical staining of the F4/80 macrophage marker in SAT and EAT biopsies revealed the decreased presence of these cells in EAT from SGLT-2i+ patients, compared with SGLT-2i− patients (Fig. 3A), which was mirrored by a decreased amount of *ADGRE1* transcripts, a gene-coding F4/80 protein, in EAT (Fig. 3B). In addition to the reduced presence of macrophages, we detected decreased levels of Th1 and Th2 lymphocytes in EAT (Fig. 3C). These results were supported by the observed overall reduction of cellularity in the EAT tissue, based on staining of nuclei using DAPI (Fig. 3A). However, we did not observe any similar changes in immune cells’ infiltration in SAT (Fig. 3A–C). These data correlate with the decreased number of transcripts of haematopoietic surface markers in EAT, compared to SAT (Fig. 1—Haematopoietic cell lineage pathway). In peripheral blood, we did not observe any changes in the levels of Th1 and Th2 lymphocytes or classic and intermediate monocytes (Suppl. Figure 1A–D). However, we detected decreased levels of immunosuppressive monocytes, an independent predictor of CAD risk (Suppl. Figure 1E).

### Effects of SGLT-2i on iron metabolism

**Fig. 4:**
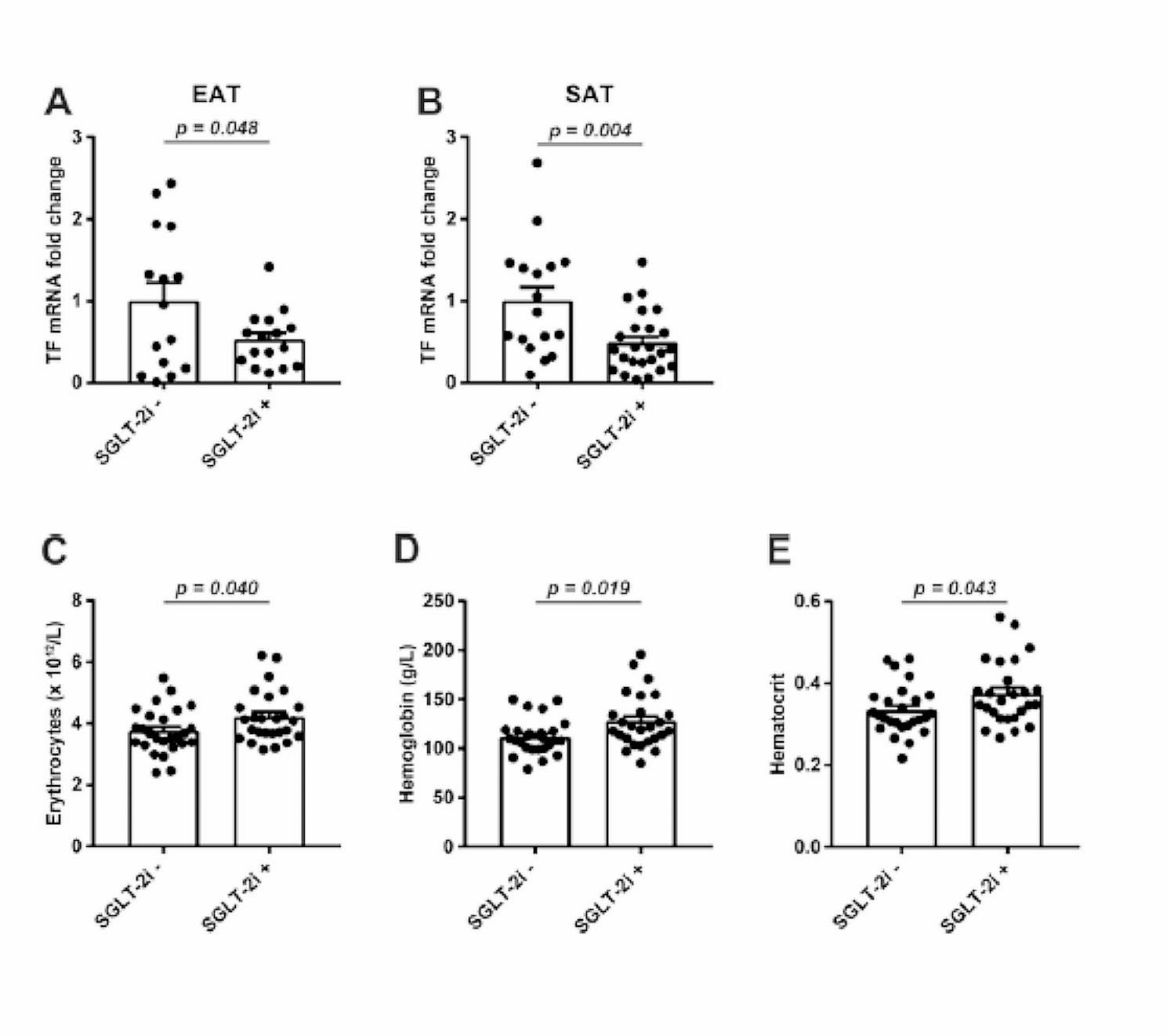
Effects of SGLT-2i on iron metabolism. Expression of *TF* gene in **A** EAT and **B** SAT of SGLT-2i− or SGLT-2i+ patients was determined by RT-qPCR (EAT: SGLT-2i− *n* = 15; SGLT-2i+ *n* = 17. SAT: SGLT-2i− *n* = 17; SGLT-2i+ *n* = 25). Number of **C** erythrocytes, **D** level of haemoglobin and **E** haematocrit were detected from whole blood of SGLT-2i− (*n* = 26) or SGLT-2i+ (*n* = 26) patients. Data are expressed as mean ± SEM

Iron metabolism is often impaired in patients with HF, which can contribute to its progression. We did not observe any changes in circulating iron (Suppl. Figure 2A), transferrin (Suppl. Figure 2B), saturated transferrin (Suppl. Figure 2C) or soluble transferrin receptor (Suppl. Figure 2D) between both groups. However, SGLT-2i+ patients showed a significant reduction in transferrin (*TF*) transcripts, a glycoprotein responsible for cellular iron transport, compared to the SGLT-2i− group in both EAT and SAT (Fig. 4A, B). The SGLT-2i+ group also had significantly higher red blood cell counts (Fig. 4C), haemoglobin (Fig. 4D), and haematocrit (Fig. 4E), confirming previously published data [28].

### SGLT-2i regulate lipid species in adipose tissue

**Fig. 5:**
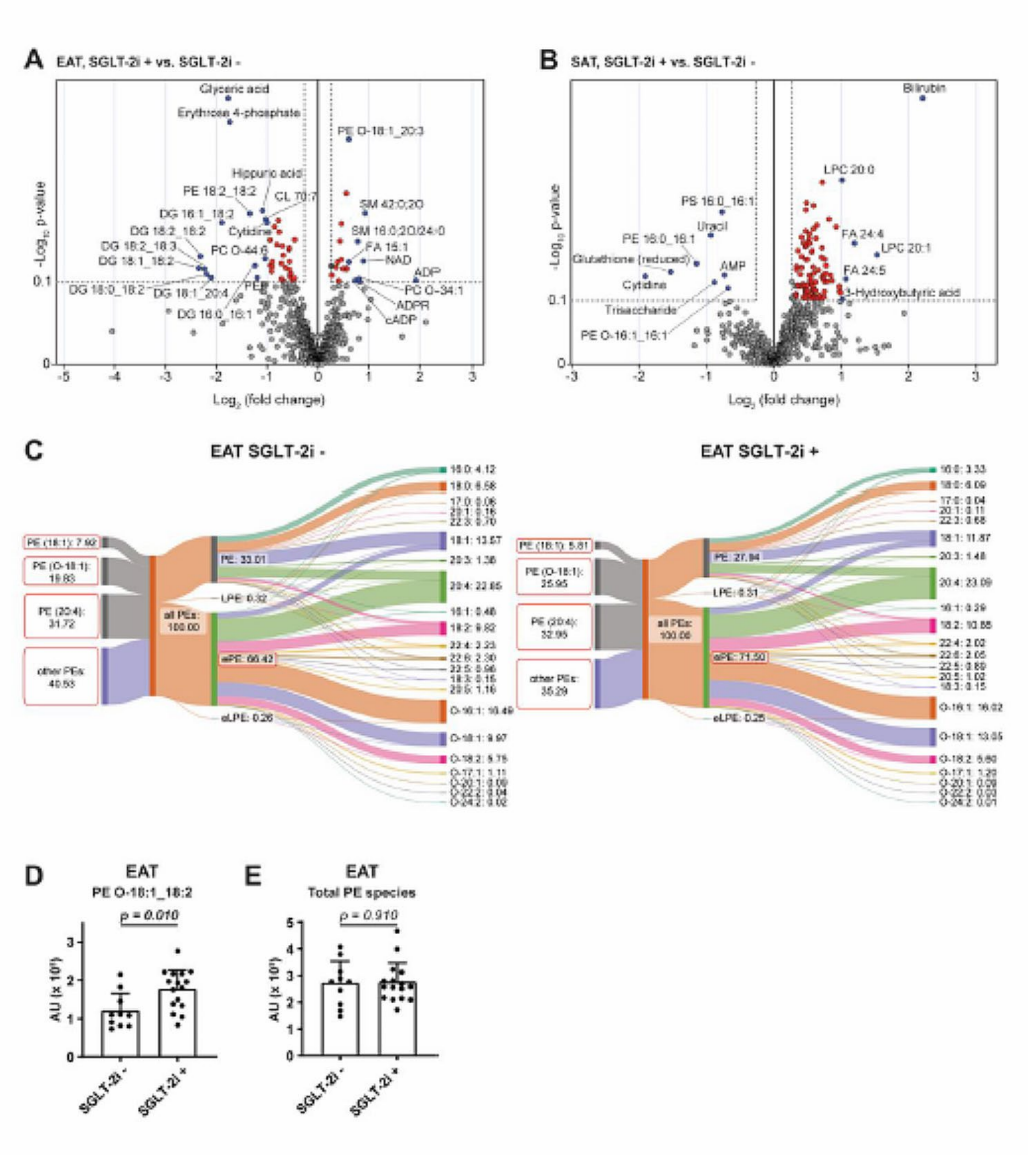
SGLT-2i regulate lipid species in adipose tissue. **A** EAT and **B** SAT metabolome contrasting SGLT-2i+ and SGLT-2i− groups (EAT: SGLT-2i− *n* = 10; SGLT-2i+ *n* = 17. SAT: SGLT-2i− *n* = 18; SGLT-2i+ *n* = 19). Dashed lines mark the area within p-value < 0.1 and fold change > 1.2 and metabolites are highlighted in red. Blue dots represent labelled metabolites with fold change > 1.5. **C** Sankey diagram of phosphatidylethanolamine (PE), lyso-phosphatidylethanolamine (LPE), and ether-phosphatidylethanolamine (ePE) species in EAT of SGLT-2i− and SGLT-2i+ groups. The left part represents the percentage of acyls 18:1, O-18:1 and 20:4 in the phosphatidylethanolamine group; the centre part represents the percentage of each class of phosphatidylethanolamine; and the right part represents the carbon chains (#carbons: #double bonds; O- ether bond), followed by the percentage value. **D** Levels of PE O-18:1_18:2 in EAT of SGLT-2i− and SGLT-2i+ groups. **E** Levels of Total PE species in EAT of SGLT-2i− and SGLT-2i + groups. Data are expressed as mean ± SEM

To complement the results from differential gene expression analysis and to identify potential metabolic traits and factors influenced by SGLT-2i, we performed metabolomic profiling of EAT and SAT (Fig. 5). Differential analysis showed that SGLT-2i treatment affected mainly lipids in both tissues (Fig. 5A, B). In EAT, it was associated with decreased levels of diacylglycerols and glycerophosphoinositols, whereas in SAT, mainly the concentrations of free fatty acids and their conjugates were increased. Although the overall effect in absolute terms was mild, the results of the over-representation analysis revealed consistent changes in ether-lipid species and carbon chains, with 18 carbon atoms in both fat depots (Tables 5 and 6).

**Table 5:** Summary of enriched lipid structural patterns in EAT

Direction	Level	Features	*p*-value	Odds ratio	FDR
Up		Ether lipids	4.71 × 10^−5^	8.51	0.17
Down	Main class	Diradylglycerols (GL02)	1.12 × 10^−4^	7.97	0.20
Down	Main class	Glycerophosphoinositols (GP06)	5.34 × 10^−4^	7.15	0.28
Down	Sub class	Diacylglycerols (GL0201)	7.43 × 10^−5^	8.56	0.27
Down	Backbone #C #DB	36:3	1.79 × 10^−4^	11.83	0.16
Down	Chain length #C	18	1.40 × 10^−4^	2.24	0.17

Untargeted metabolomic and lipidomic profiling was performed on samples of EAT from subjects treated with either SGLT-2i− (*n* = 10) or SGLT-2i+ (*n* = 17). Data with false discovery rate (FDR) < 0.3 are presented

#C, number of carbons; #DB, number of double bonds

**Table 6:** Summary of enriched lipid structural patterns in SAT

Direction	Level	Features	*p*-value	Odds ratio	FDR
Up		Fatty acids (FA)	1.72 × 10^−9^	5.48	0.00
Up	Main class	Fatty acids and conjugates (FA01)	4.25 × 10^−6^	5.03	0.00
Up	Main class	Glycerophosphocholines (GP01)	6.18 × 10^−4^	2.41	0.28
Up	Sub class	1-alkyl, 2-acylglycerophosphocholines (GP0102)	5.19 × 10^−6^	4.25	0.00
Up	Sub class	Fatty acyl carnitines (FA0707)	3.40 × 10^−4^	4.84	0.21
Up	Sub class	Unsaturated fatty acids (FA0103)	4.25 × 10^−6^	5.03	0.00
Up	Chain length #C	18	2.83 × 10^−7^	8.51	0.00
Up		Ether lipids	5.33 × 10^−4^	2.50	0.28
Down	Backbone	32:1	4.28 × 10^−4^	175.08	0.26
Down	Acyls	16:1	3.18 × 10^−4^	27.71	0.29
Down	Acyl length	16	5.73 × 10^−5^	12.55	0.07

Untargeted metabolomic and lipidomic profiling was performed on samples of SAT from subjects treated with either SGLT-2i− (*n* = 18) or SGLT-2i+ (*n* = 19). Data with false discovery rate (FDR) < 0.3 are presented

#C, number of carbons

SGLT-2i treatment was associated with an increased proportion of ether-phosphatidylethanolamines (ePE: 71.50% in SGLT-2i+ group vs. 66.42% in SGLT-2i− group) in EAT (Fig. 5C), especially in PE containing ether-linked oleic acid (PE O-18:1) at the expense of ordinary acyl PEs (PE other: 35.29% in SGLT-2i+ group vs. 40.53% in SGLT-2i− group) and PE containing oleic acid (PE 18:1: 5.81% in SGLT-2i+ group vs. 7.92% in SGLT-2i− group). The marker lipid of this shift, PE O-18:1_18:2 (Fig. 5D), was significantly increased in EAT from the SGLT-2i+ group, while the levels of ether-PEs containing pro-inflammatory arachidonic acid (20:4 acyls) were almost unchanged (32.95% in SGLT-2i+ group vs. 31.72% in SGLT-2i− group), as were the levels of total PEs (Fig. 5C, E). This strong effect of SGLT-2i was not observed in SAT, where the most PEs were almost unchanged. However, a slight increase in ether-PEs content was detected in SAT (ePE: 70.63% in SGLT-2i+ group vs. 66.97% in SGLT-2i− group) (Suppl. Figure 3 A).

## Discussion

Modification of EAT was recently postulated [48] as one of the potential mechanisms by which SGLT-2i might exert their beneficial effects on HF. Here we show that SGLT-2i treatment in subjects with HF with severely reduced ejection fraction was in EAT associated with (a) shift in gene expression towards reduced synthesis and increased degradation of fatty acids and decreased pro-inflammatory, programmed cell death and senescence traits, (b) reduction of immune cell, especially macrophage, content and (c) lipid remodelling and enrichment with anti-inflammatory lipid species.

SGLT-2i treatment is associated with significant effects on adipose tissue metabolism, characterised by activation of lipolysis and fatty acid oxidation, with a resulting increase in ketone bodies’ synthesis due to elevated glucagon levels from glycosuria [49]. This increase in ketone bodies is proposed as a potential cardioprotective mechanism of SGLT-2i. Considering the proximity of epicardial adipose tissue (EAT) to the myocardium, increased ketone body production in EAT may directly influence myocardial tissue, improving its energetic metabolism [50]. Here, we did not find any significant difference in the levels of the main ketone body, 3hydroxybutyrate, between SGLT-2i+ and SGLT-2i− groups in either EAT or SAT, challenging the role of EAT-derived ketone bodies in SGLT-2i- associated cardioprotection in heart failure with reduced ejection fraction (HFrEF). However, the notably elevated level of 3-hydroxybutyric acid in EAT compared to SAT in the SGLT-2- group still suggests stimulation of ketogenesis in EAT as a potential cardioprotective reaction in severe HF. With increased concentrations of 3-hydroxybutyric acid observed in SAT, but not in EAT, under SGLT-2i treatment, this difference is eliminated, indicating an adipose tissue depot-specific effect of SGLT-2i on ketogenesis, favouring SAT. One might speculate that, in severe HF, EAT-derived ketone bodies’ production is already at its peak and thus SGLT2i treatment is not able to increase it any further, whereas SAT with a markedly lower production than EAT still has reserves that can be exploited by SGLT2i administration. However, this hypothesis requires further proof in dedicated molecular studies.

Gene expression analysis related to lipid metabolism in our severe HF cohort indicates that EAT and SAT exhibit similar gene expression patterns after SGLT-2i treatment, but a stronger effect was observed in EAT. SGLT-2i treatment has been associated with a reduction in EAT thickness in previous studies [51], consistent with increased activation of lipolysis observed in our gene expression analysis. Although this finding may seem somewhat paradoxical, considering the reported positive association between HFrEF and reduced EAT thickness, it could suggest that mechanisms other than their lipolytic effect on EAT play a more significant role in the cardioprotective effects of SGLT-2i. EAT and myocardium may have a similar response to SGLT-2i treatment. A recent study [52] showed that cardiomyocytes produce SGLT-2, and its expression increases in patients with type 2 diabetes mellitus. Another study [53] observed that diabetic patients who underwent heart transplantation have significantly higher, even lipotoxic, levels of lipids and ceramides in the heart and that SGLT-2i treatment was associated with a reduction in both TAG and ceramides. In patients with severe HF, SGLT-2i are likely to have a direct effect on cardiomyocytes, particularly in reducing lipotoxic lipid levels and thus mitigating parameters of HF [53]. The increase in fatty acid degradation and reduction of programmed cell death and senescence traits seen in our study seem to complement these effects on cardiomyocytes, suggesting a synergistic and supportive influence of SGLT2i on both myocardium and adjacent EAT.

Adipose tissue inflammation is one of the key processes responsible for the development of obesity-associated complications including insulin resistance, T2DM and accelerated atherosclerosis, all of which are established risk factors for HF. Under physiological conditions, EAT shows an anti-inflammatory phenotype with predominant secretion of anti-inflammatory and cardioprotective adipo- and cytokines such as adiponectin, omentin, apelin and others [54, 55]. Expansion of EAT associated mostly with visceral obesity was shown to change its secretome to pro-inflammatory with the reduction in adiponectin and increased production of pro-inflammatory cytokines including TNFα, IL1β, IL6, Monocyte Chemoattractant Protein-1, TGFβ and others [56]. Other studies have shown increased levels of pro-inflammatory factors in the EAT of patients with heart failure, which may lead to cardiomyocyte apoptosis, increased fibroblast activation and subsequent fibrosis, contributing to myocardial dysfunction [25, 57, 58] and the possibility of systolic HF [59]. Here, our gene expression analysis showed that, in both EAT and SAT subjects, SGLT-2i treatment was associated with a reduction in most inflammation-related pathways, including NF-κB, JAK/STAT and adipokine pathways, as well as senescence, cell cycle and apoptosis regulating traits, all key intersections between metabolic and immune inflammatory signalling. This is consistent with the results of Díaz-Rodríguez et al., who demonstrated reduced secretion of pro-inflammatory chemokines in EAT explants treated with SGLT-2i dapagliflozin [48], which was also confirmed in experimental settings [30, 60–63].

CAD was described as being associated with increased macrophage content in EAT and their polarisation towards the pro-inflammatory M1 phenotype, which enhances the inflammation and worsens the course of disease [64, 65]. Moreover, subjects with HFpEF exerted an M1 pro-inflammatory macrophage phenotype in a study by Patel et al. [66]. In experimental animals, SGLT-2i reduced macrophage infiltration and M1 polarisation in visceral and perivascular adipose tissue, as well as in cardiomyocytes in an ischaemic heart rodent model [60, 67–69]. In a study by Sardu et al., SGLT-2i therapy was associated with a significant reduction of coronary plaque thickness and macrophage infiltration and an increase in plaque stability, along with amelioration of systemic inflammatory burden in patients with T2DM and multivessel non-obstructive coronary stenosis, resulting in a reduction of cardiovascular events [70]. Here, we show increased macrophage infiltration in EAT in subjects without SGLT-2i treatment, which was substantially less present in the SLGT2-i+ group. Interestingly, this decrease was not seen in SAT, which is in agreement with previous findings [60].

Accumulation of T lymphocytes in adipose tissue was suggested to initiate macrophage recruitment into adipose tissue in obesity [71]. A recent work by Zhang et al. showed that EAT of HF patients undergoing heart transplantation was characterised by an accumulation of T cells, especially INFγ^+^ effector T cells [72]. Here, FACS analysis has demonstrated a reduced percentage of both Th1 and Th2 cells in EAT, but not in SAT. Overall, our data suggest for the first time in humans that SGLT-2i treatment might be associated with the reduction of inflammatory immune cell content, specifically in EAT, thus highlighting one of the possible traits contributing to the cardioprotective effects of SGLT-2i.

Metabolites serve as direct representatives of biochemical activity and can be more easily correlated with the underlying phenotypes. Therefore, we tried to evaluate the influence of SGLT-2i treatment on the metabolomic profile of both EAT and SAT. In line with the gene expression data and the effect of SGLT-2i on lipid metabolism, the main changes were seen in the lipid spectrum. Previously, lipidomic profiling showed significant changes in EAT as compared to SAT, with increased levels of phospholipid species (including phosphatidylcholines and phosphatidylethanolamines) and sphingolipids (sphingomyelins) [73, 74]. Also, CAD was associated with higher levels of ceramides and atherogenic ceramide ratios as well as di- and monoacylglycerols, while unsaturated triacylglycerols were reduced [74, 75]. In advanced HF, cardiac cachexia was associated with an increased amount of the phospholipid cardiolipin in EAT [76]. In our subjects with advanced HFrEF, SAT of the SGLT-2i+ group showed elevated levels of fatty acids and their conjugates, along with fatty acid carnitines, their transporters into mitochondria. In addition, the main ketone body, 3-hydroxybutyric acid, was elevated, confirming the primary lipolytic and β-oxidation- and ketogenesis-promoting effect of SGLT-2i in SAT. In contrast, no such effect was seen in EAT. Instead, EAT of SGLT-2i subjects was characterised by reduced diacylglycerols and glycerophosphoinositols, rendering it possibly less proatherogenic, given their aforementioned positive association with CAD [74]. Moreover, significantly increased levels of ether lipids were noted in EAT from the SGLT-2i+ group, especially in ePE with 18 carbon atoms. This increase was driven mainly by the elevated migration of oleic acid to the ether form PE O-18:1. On the other hand, the pro-inflammatory arachidonic acid was unchanged in its representation in PE. The observed shift in the overall PE profile, especially in ePE species, suggests a change in the composition of cell membranes that may be undergoing more dynamic movement [77]. This positive change in cellular membranes indicates the reduction of ferroptosis, an iron-dependent programmed cell death pathway, different from other programmed cell death types such as apoptosis, necrosis, pyroptosis or autophagy [78]. Ferroptosis is defined as an iron-dependent form of regulated cell death that involves the iron-catalysed accumulation of lethal lipid peroxides and is thus closely connected with lipid peroxidation and changes in the spectrum of phospholipid species [79–82]. The iron-carrier protein transferrin and its receptor have been shown to be essential for the development of ferroptosis [83]. Transferrin delivers iron into the cells by binding to the transferrin receptor and the whole complex is then internalised by endocytosis into the cytoplasm of the cell, where Fe^3+^ is reduced to Fe^2+^ and released [84, 85]. Thus, the reduction of transferrin mRNA level seen in our study further supports the anti-ferroptotic effect of SGLT-2i treatment.

Recently, ferroptosis has been implicated in the pathogenesis of CAD, including myocardial infarction, ischaemia/reperfusion injury, cardiomyopathies (including diabetic cardiomyopathy), and HF [86–88]. In addition, treatment with SGLT-2i was associated with a reduction in ferroptosis in experimental models of doxorubicin-induced cardiomyopathy and ischaemia/reperfusion injury [61, 89]. Reduction in ferroptosis in EAT might thus be another potential trait by which SGLT-2i ameliorate its pro-inflammatory state, which then further translates into improved myocardial function, although this hypothesis requires further proof.

## Conclusion

In conclusion, in the present work we have demonstrated that in subjects with severe HF with reduced ejection fraction, treatment with SGLT-2i is associated with reduced inflammation and pro-inflammatory immune cell infiltration and an anti-inflammatory and anti-ferroptotic shift in the lipid spectrum in EAT, but not SAT. These mechanisms might at least partially explain the beneficial effects of SGLT-2i in HFrEF.

### Electronic supplementary material

Below is the link to the electronic supplementary material.

Supplementary Figure 1. Presence of (A) Th1 lymphocytes, (B) Th2 lymphocytes, (C) classic monocytes, (D) intermediate monocytes and (E) immunosuppressive monocytes in whole blood of SGLT-2i– (*n* = 24) and SGLT-2i + (*n* = 9) patients was determined by FACS analysis. Data are expressed as mean ± SEM.

Supplementary Figure 2. Levels of iron (A), transferrin (B) saturated transferrin (C) and soluble transferrin receptor (sTfR) (D) were detected from whole blood of SGLT-2i– (*n* = 26) and SGLT-2i + (*n* = 26) patients. Data are expressed as mean ± SEM.

Supplementary Figure 3. (A) Sankey diagram of phosphatidylethanolamine (PE), lyso-phosphatidylethanolamine (LPE), and ether-phosphatidylethanolamine (ePE) species in SAT of SGLT-2i– (*n* = 18) and SGLT-2i + (*n* = 19) groups. The left part represents the percentage of acyls 18:1, O-18:1 and 20:4 in the phosphatidylethanolamine group; the centre part represents the percentage of each class of phosphatidylethanolamine; and the right part represents the carbon chains (#carbons: #double bonds; O- ether bond) followed by the percentage value.

## Data Availability

The data presented in this study are available on request from the corresponding author.

## Abbreviations

#C: Number of carbons
#DB: Number of double bonds
ACTB: β-actin
ADGRE1: Adhesion G protein-coupled receptor E1
ADPR: Adenosine 5′-diphosphoribose
ALT: Alanine aminotransferase
AST: Aspartate aminotransferase
BMI: Body mass index
BNP: Brain natriuretic peptide
CAD: Coronary artery disease
cADP: Cyclic adenosine diphosphate ribose
CCL2: Chemokine (C–C motif) ligand 2
CRP: C-reactive protein
CSF2RA: Colony stimulating factor 2 receptor, alpha, low-affinity (granulocyte-macrophage)
CUDA: 12-[(Cyclohexylamino)carbonyl]amino]-dodecanoic acid
CXCL2: Chemokine (C–X–C motif) ligand 2
CXCR4: Chemokine (C–X–C motif) receptor 4
DAPI: 4′,6-Diamidino-2-phenylindole
DG: Diacylglycerol
DM: Diabetes mellitus
EAT: Epicardial adipose tissue
EF: Ejection fraction
ePE: Ether-phosphatidylethanolamine
FA: Fatty acid
FACS: Flow cytometry
FDR: False discovery rate
HbA_1c_: Haemoglobin A_1c_
HDL: High-density lipoprotein
HF: Heart failure
HFpEF: Heart failure with preserved ejection fraction
HFrEF: Heart failure with reduced ejection fraction
IKEM: Institute for Clinical and Experimental Medicine
IGF1: Insulin like growth factor 1
IL1β: Interleukin 1β
IL1R1: Interleukin 1 receptor type 1
IL1RAP: Interleukin 1 receptor accessory protein
IL6: Interleukin 6
IL8: Interleukin 8
IL10: Interleukin 10
INFγ: Interferonγ
JAK/STAT: Janus kinase/signal transduction and transcription activation
KEGG: Kyoto Encyclopaedia of Genes and Genomes
LC–MS: Liquid chromatography–mass spectrometry
LDL: Low-density lipoprotein
LPC: Lyso-phosphatidylcholine
LPE: Lyso-phosphatidylethanolamine
LV: Left ventricular
LVEDd: Left ventricular end-diastolic diameter
LV EF: Left ventricular ejection fraction
NF-κB: Nuclear Factor κB
NYHA: New York Heart Association
PBS: Phosphate buffered saline
PC: Phosphatidylcholine
PE: Phosphatidylethanolamine
PEP: Phosphoenolpyruvic acid
PS: Phosphatidylserine
SAT: Subcutaneous adipose tissue
SEM: Standard error of mean
sTfR: Soluble transferrin receptor
SGLT-2: Sodium-glucose cotransporter 2
SGLT-2i: Sodium-glucose cotransporter 2 inhibitors
SGLT-2i−: Subjects without SGLT-2i treatment
SGLT-2i+: Subjects with SGLT-2i treatment
SM: Sphingomyelin
T2DM: Type 2 diabetes mellitus
TF: Transferrin
TGFβ: Transforming growth factor β
TNFα: Tumour Necrosis Factor α
TNFAIP3: TNFα induced protein 3
TRAF1: TNF receptor associated factor 1
WNT: Wingless-related
