# Supplementary figures and images for: Sodium-glucose cotransporter 2 inhibitors induce anti-inflammatory and anti-ferroptotic shift in epicardial adipose tissue of subjects with severe heart failure
Source: Cardiovasc Diabetol. 2024 Jun 28;23:223. doi: 10.1186/s12933-024-02298-9 (PMC11214218; doi:10.1186/s12933-024-02298-9)

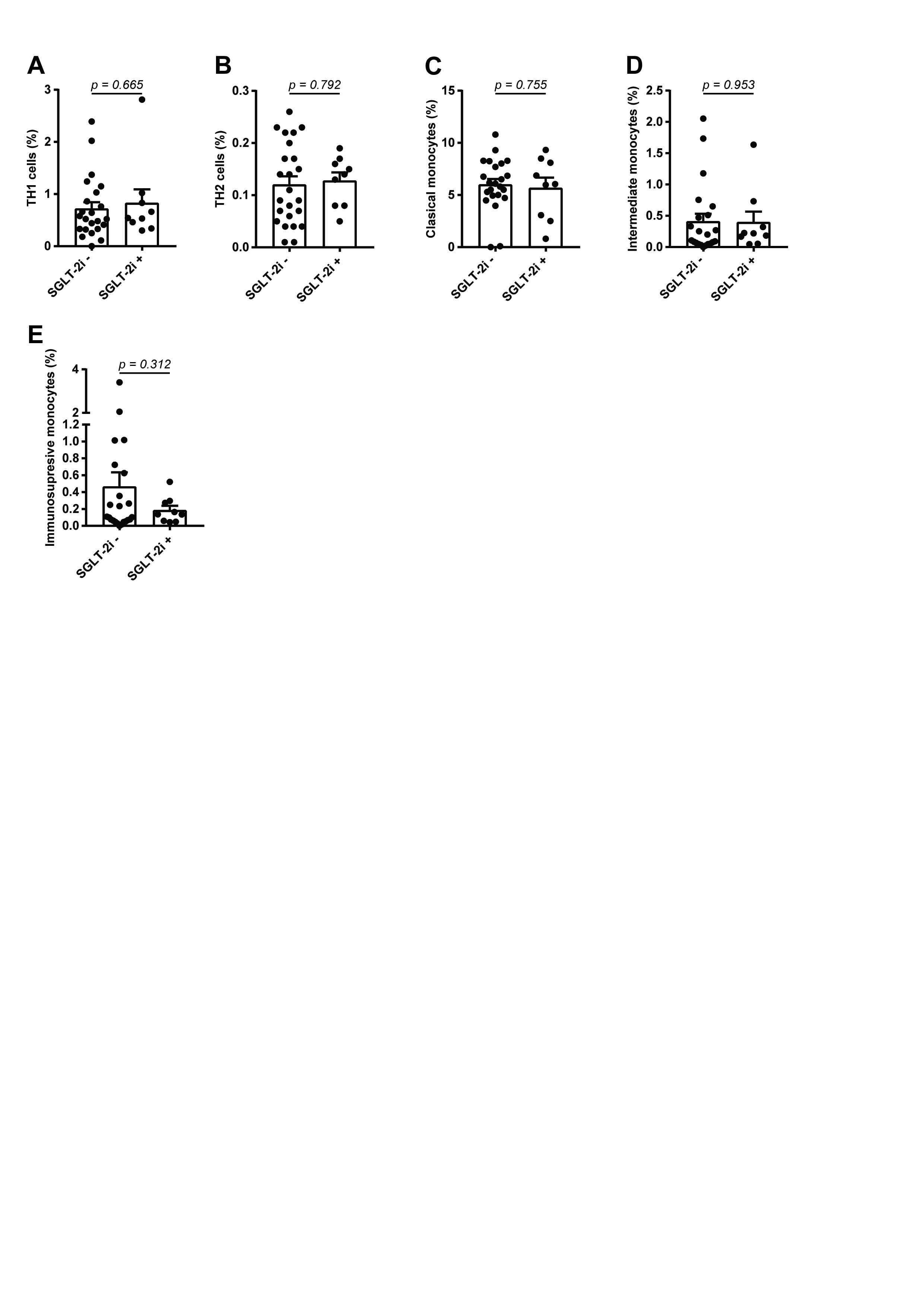

Supplement: Supplementary file 1 — Supplementary Figure 1. Presence of (A) Th1 lymphocytes, (B) Th2 lymphocytes, (C) classic monocytes, (D) intermediate monocytes and (E) immunosuppressive monocytes in whole blood of SGLT-2i– (n = 24) and SGLT-2i + (n = 9) patients was determined by FACS analysis. Data are expressed as mean ± SEM. [file 12933_2024_2298_MOESM1_ESM.tif]

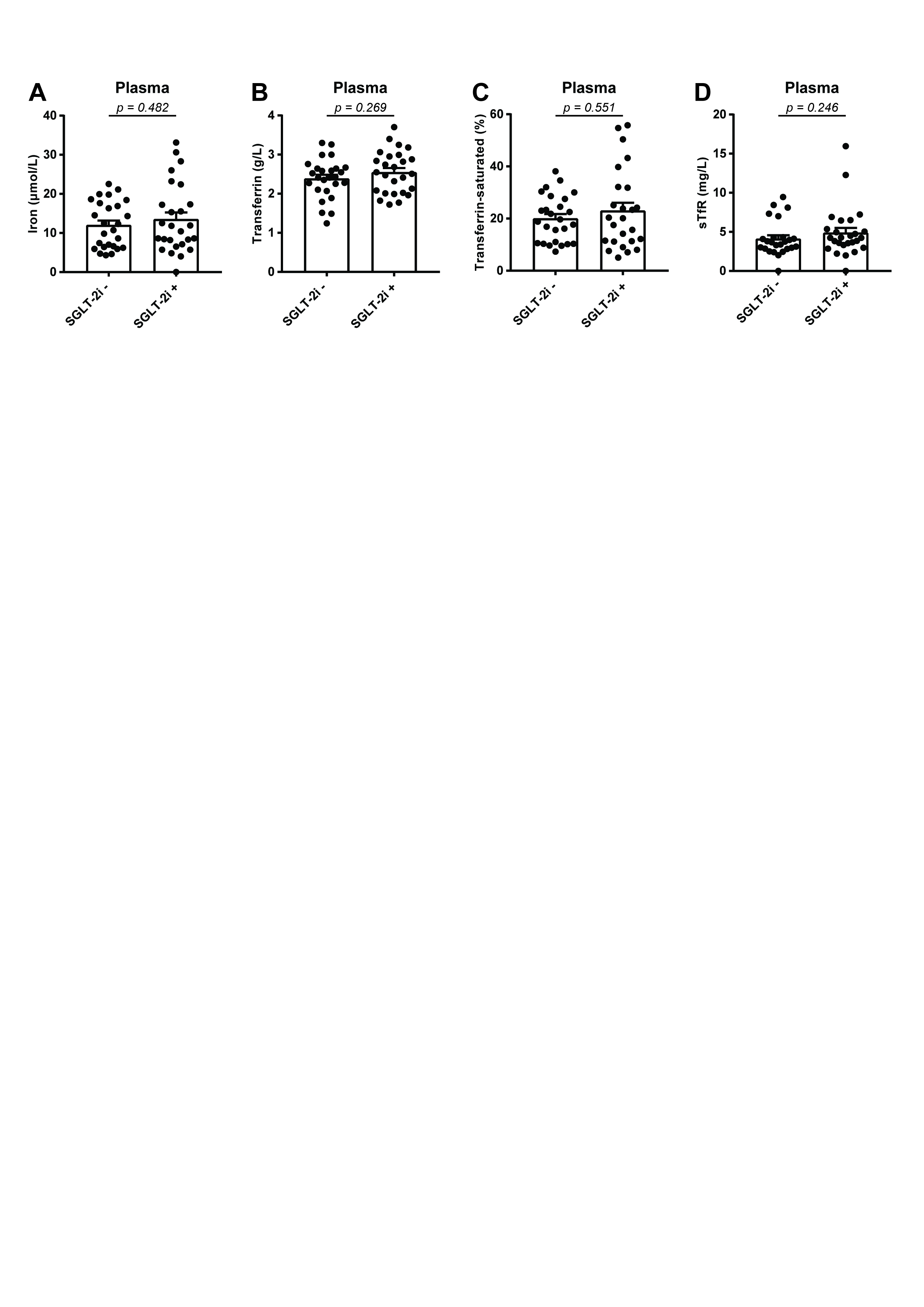

Supplement: Supplementary file 2 — Supplementary Figure 2. Levels of iron (A), transferrin (B) saturated transferrin (C) and soluble transferrin receptor (sTfR) (D) were detected from whole blood of SGLT-2i– (n = 26) and SGLT-2i + (n = 26) patients. Data are expressed as mean ± SEM. [file 12933_2024_2298_MOESM2_ESM.tif]

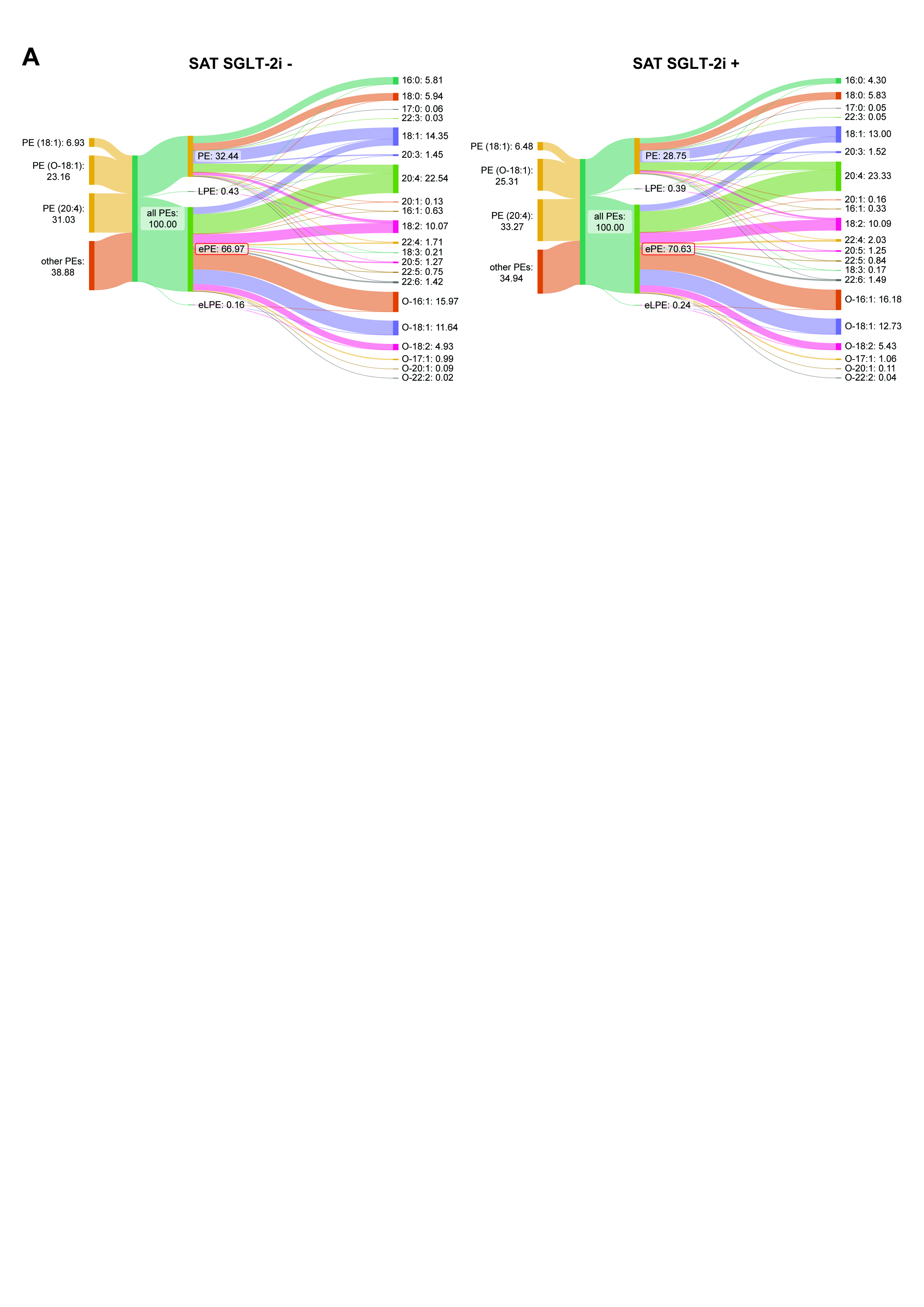

Supplement: Supplementary file 3 — Supplementary Figure 3. (A) Sankey diagram of phosphatidylethanolamine (PE), lyso-phosphatidylethanolamine (LPE), and ether-phosphatidylethanolamine (ePE) species in SAT of SGLT-2i– (n = 18) and SGLT-2i + (n = 19) groups. The left part represents the percentage of acyls 18:1, O-18:1 and 20:4 in the phosphatidylethanolamine group; the centre part represents the percentage of each class of phosphatidylethanolamine; and the right part represents the carbon chains (#carbons: #double bonds; O- ether bond) followed by the percentage value. [file 12933_2024_2298_MOESM3_ESM.tif]
